# Postoperative complications and hospital costs following elective and emergency colon cancer surgery: a Clavien-Dindo grade-specific cost analysis

**DOI:** 10.1007/s00384-026-05252-1

**Published:** 2026-10-13

**Authors:** Sandra Bech-Larsen, Tomas Vedin, Jenny Engdahl, Astrid Öberg, Stefan Öberg

**Affiliations:** 1 Department of Surgery, Helsingborg Hospital and Clinical Sciences Lund, Lund University, S-251 87 Helsingborg, Sweden; 2 Department of Surgery, Skåne University Hospital and Clinical Sciences Malmö, Lund University, Malmö, Sweden

**Keywords:** Colon cancer surgery, Hospital costs, Clavien-Dindo classification, Health economic analysis, Healthcare costs, Postoperative complications

## Abstract

**Purpose:**

Postoperative complications are drivers of healthcare costs following colon cancer surgery. We compared hospital costs between elective and emergency colon cancer resections according to complication severity.

**Methods:**

This retrospective cohort study included 520 patients undergoing colon cancer resection, of whom 431 underwent elective and 89 emergency surgery. Postoperative complications were graded according to the Clavien-Dindo classification. Hospital costs were analysed using a micro-costing approach. Adjusted costs were estimated using generalized linear models with gamma distribution and log link, adjusting for tumour stage, ASA classification, age and sex, with surgical technique in elective surgery.

**Results:**

CD II–V occurred in 35% of elective and 56% of emergency patients. Emergency patients had higher ASA class, more advanced tumour stage and lower BMI (*p* < 0.05). Among patients with no or minor complications (CD 0–I), median costs were €9809 following elective surgery and €12,079 following emergency surgery. Among patients with complications, costs were higher in emergency versus elective surgery (€19,655 vs. €14,820; *p* = 0.004). Adjusted excess costs increased from CD 0–I to CD IV, whereas CD V did not follow this pattern. In adjusted analyses, complication severity remained associated with increased costs, with cost ratios of 3.21 in elective and 4.86 in emergency surgery for grade IV vs no or minor complications; however, the emergency estimate was based on six patients with CD IV complications and should be interpreted cautiously.

**Conclusions:**

Complication severity was independently associated with substantial cost increases after colon resection. Observed costs were generally higher and more variable following emergency surgery.

**Supplementary Information:**

The online version contains supplementary material available at https://doi.org/10.1007/s00384-026-05252-1.

## Introduction

Colorectal cancer is the third most common malignancy in Sweden with more than 7000 new cases diagnosed annually, placing a substantial burden on healthcare systems and hospital resources. In Sweden, approximately 11% of colon cancer resections are performed in the emergency setting. Postoperative complication rates after colon cancer surgery have remained relatively stable over the past two decades, affecting approximately 20% of elective cases, whereas rates are approximately doubled in emergency surgery [1].

Postoperative complications constitute major drivers of healthcare costs following colorectal cancer surgery, with higher costs generally observed with increasing complication severity [2]. This burden is further accentuated in emergency colon resections, where patients present with more advanced disease and a significantly higher risk of postoperative complications, reoperations, and mortality compared with elective surgery [1]. Pooling elective and emergency resections may obscure important differences in perioperative risk, postoperative resource utilization, and cost drivers between these fundamentally different clinical settings.

Despite this, most previous cost analyses have either pooled elective and emergency colon resections [3, 4], or included heterogeneous surgical populations across different surgical specialties [5, 6], limiting interpretation of procedure-specific cost drivers. Furthermore, substantial heterogeneity exists in healthcare systems, reimbursement structures, costing methodologies, and time horizons, limiting direct comparison between published cost estimates [7]. Although several studies have evaluated costs associated with specific postoperative complications, such as surgical site infections or anastomotic leakage, patients frequently experience multiple concurrent complications. Therefore, overall complication severity may better reflect the postoperative resource utilization than isolated adverse events. To our knowledge, no previous study has conducted an adjusted grade-specific cost analysis across Clavien-Dindo grades with separate analyses for elective and emergency surgery.

The aim of this study was therefore to conduct a detailed cost analysis of colon cancer surgery, with separate analyses of elective and emergency resections, and to quantify excess costs associated with postoperative complications stratified by Clavien-Dindo grade [8], using a generalized linear model.

## Method

### Study population and complications

We conducted a single-centre retrospective health economic study of patients aged >18 years who underwent colon resection for colon cancer at Helsingborg General Hospital between 2016 and 2022. The study size was determined by the number of consecutive patients meeting the eligibility criteria during the predefined study period. The study period was predefined based on the availability of consistent patient-level hospital cost data. The Swedish Colorectal Cancer Registry (SCRCR) was used to identify eligible patients and to extract demographic and clinical data. The SCRCR is a prospective national quality registry for colorectal cancer with near-complete national coverage [1, 9]. The medical records of each patient were reviewed to validate registry data, assess postoperative complications within 30 days, and obtain additional clinical information, including whether patients had received neoadjuvant or palliative chemotherapy for colon cancer. No predefined time interval relative to surgery was applied. Only patients undergoing colon resection for colon cancer were included, patients undergoing colon resections for benign disease, rectal or appendiceal cancer, or isolated stoma formation without concomitant tumour resection were excluded. Both elective and emergency cancer resections were included and analysed separately. Emergency surgery was defined as unplanned colon resections performed within 48 h of acute hospital admission, whereas all other resections were defined as elective procedures.

Postoperative complications were classified according to the Clavien-Dindo classification [8, 10]. In patients with multiple complications, the complication with the highest Clavien-Dindo grade was used. Patients with Clavien-Dindo grades 0–I were categorized as having no or minor complications, as minor deviations from the normal postoperative course are difficult to reliably identify retrospectively and are unlikely to substantially influence hospital costs or clinical outcomes. For analyses according to complication type, postoperative complications were categorized as cardiac, non-surgical-site infection (non-SSI), or surgical complications. Cardiac complications included postoperative cardiac events such as atrial fibrillation, heart failure, and myocardial infarction. Non-SSI complications were defined as infectious complications not related to the surgical site, such as pneumonia and urinary tract infection. Surgical complications were defined as complications directly related to the surgical procedure, including surgical site infections, intra-abdominal abscesses, anastomotic leakage, postoperative ileus, renal failure and other procedure-related complications. These three categories were not considered mutually exclusive or exhaustive of all postoperative complications. Thromboembolic complications were not analysed as a separate category because the eight patients with deep vein thrombosis or pulmonary embolism also had a cardiac, non-SSI, or surgical complication and were therefore included within these predefined categories instead.

### Cost analysis

Subsequently, we performed an analysis of the hospital costs associated with different types of complications according to the Clavien-Dindo classification. Total in-hospital costs were obtained and analysed in collaboration with a health economist. Costs reflected actual hospital expenditures derived from the institutional cost accounting system rather than reimbursement data or hospital charges.

The costs were categorized into the following components: operating room, anaesthesia, postoperative unit care, intensive care unit (ICU), laboratory and radiologic services, medications and medical supplies, staff salaries, and other costs. The category of “other costs” included premises-related expenses and emergency department utilization.

A patient-level micro-costing approach was used [11]. Costs were calculated for the entire clinically related hospital care episode attributable to the surgical treatment and postoperative course. This included the index admission and, when applicable, subsequent readmissions considered clinically related to the index surgery. The clinical relatedness of subsequent admissions was assessed by the study group through review of the medical records, based on the documented clinical relationship to the index surgical episode. The cost analysis was therefore not restricted to the 30-day postoperative period used for complication ascertainment but included all direct hospital costs incurred during the clinically related care episode. There was no predefined maximum time window for inclusion of clinically related readmissions. The exact duration of cost accrual from the index surgery to the final cost-bearing hospital encounter could not be determined from the available cost data, as costs were recorded at the level of complete hospital admissions rather than individual hospital days. All cost components were assigned on a per-patient basis using the hospital’s cost accounting system. Only direct healthcare costs were included in the analysis, while indirect costs, such as productivity losses, were excluded.

Excess costs associated with increasing Clavien-Dindo grades were calculated, using patients with no or minor postoperative complications as the reference group.

### Statistical analysis

Given the skewed distribution of cost data, non-parametric methods were applied for descriptive analyses, and results were presented as medians with interquartile ranges (Q1–Q3). Categorical variables were presented as numbers and percentages and comparisons of proportions were made using the chi-square test. Comparisons of continuous variables were made using the Mann–Whitney *U* test for two groups and the Kruskal-Wallis test for more than two groups.

To estimate adjusted costs and excess costs, a generalized linear model (GLM) [12] with gamma distribution and log link was used. For elective surgery, the models were adjusted for tumour stage, ASA classification, surgical technique, sex and age, whereas surgical technique was not included in the emergency surgery model because only three patients underwent laparoscopic surgery. There were no missing data for variables included in the multivariable analyses. The scale parameter was estimated using maximum likelihood, and robust covariance estimates were used to obtain robust standard errors, confidence intervals and *p* values. As a sensitivity analysis, year of surgery was additionally included in the multivariable models to account for potential secular trends during the study period. Because inclusion of year of surgery did not materially change the estimated cost ratios or the study conclusions, results from the more parsimonious model were reported. Gamma distribution with log link was selected due to the right-skewed and non-negative nature of healthcare cost data. The results were presented as estimated marginal means with 95% confidence intervals, as gamma generalized linear models estimate arithmetic mean costs despite skewed cost distributions. Confidence intervals for excess costs were derived from pairwise comparisons of estimated marginal means using the Wald method, using the no or minor complication group as the reference category. Separate models were constructed for elective and emergency surgery. In analyses according to complication type, cardiac, non-SSI and surgical complications were included simultaneously as separate covariates in the same multivariable model to estimate the independent cost associated with each complication category.

All costs were adjusted to 2024 price levels using the Swedish Consumer Price Index (CPI) [13], based on annual average CPI values, and subsequently converted from SEK to Euros using the 2024 annual average EUR/SEK exchange rate (1 EUR = 11.43 SEK) [14].

Statistical analysis was performed using the Statistical Package for Social Sciences (IBM SPSS, version 31.0) and a *p* value < 0.05 was considered statistically significant.

The study was approved by the Swedish Ethical Review Authority (2019–04329). The study was reported in accordance with the Strengthening the Reporting of Observational Studies in Epidemiology (STROBE) recommendations.

## Results

### Participants and complications

A total of 520 patients were included, of whom 431 underwent elective resections and 89 underwent emergency resections. Postoperative complications occurred in 151 elective patients (35%) and 50 emergency patients (56%) (Table 1).

**Table 1:** Baseline and perioperative characteristics of patients undergoing elective and emergency colon surgery

	Elective surgery, *n* = 431 (82.9)	Emergency surgery, *n* = 89 (17.1)	*p* value
Emergency indication			
Bowel obstruction	-	73 (82.0)	-
Perforation		12 (13.5)	
Bleeding		4 (4.5)	
Clavien-Dindo classification			
No or minor complications	280 (65.0)	39 (43.8)	*p* < 0.001
(CD 0–I)	114 (26.5)	31 (34.8)	
II	21 (4.9)	5 (5.6)	
III	11 (2.6)	6 (6.7)	
IV	5 (1.2)	8 (9.0)	
V			
Age (years)	74 (68–80)	74 (67–84)	*p* = 0.915
Sex ratio (M:F)	207 (48.0): 224 (52.0)	47 (52.8): 42 (47.2)	*p* = 0.411
Body mass index	26.3 (23.9–29.7)	24.0 (21.8–27.8)	*p* = 0.018
ASA I	61 (14.2)	12 (13.5)	*p *= 0.008
ASA II	234 (54.3)	37 (41.6)	
ASA III	131 (30.4)	35 (39.3)	
ASA IV	5 (1.2)	5 (5.6)	
Tumour stage I	103 (23.9)	2 (2.2)	*p* < 0.001
Tumour stage II	179 (41.5)	32 (36.0)	
Tumour stage III	136 (31.6)	42 (47.2)	
Tumour stage IV	13 (3.0)	13 (14.6)	
Chemotherapy for colon cancer	10 (2.3)	7 (7.9)	*p* = 0.007
Surgical technique			
Open	308 (71.5)	86 (96.6)	*p* < 0.001
Laparoscopic	123 (28.5)	3 (3.4)	
Surgical procedure			
Right-sided colectomy	231 (53.6)	45 (50.6)	*p* = 0.124
Transverse colectomy	11 (2.6)	2 (2.2)	
Left-sided colectomy	47 (10.9)	11 (12.4)	
Sigmoid colectomy	129 (29.9)	23 (25.8)	
Subtotal colectomy	13 (3.0)	8 (9.0)	
Stoma formation			
Any stoma	38 (8.8)	38 (42.7)	*p* < 0.001
End colostomy	32 (7.4)	36 (40.4)	*p* < 0.001
Diverting stoma	6 (1.4)	2 (2.2)	*p* = 0.551
Length of stay (days)	8 (6–11)	10 (8–19)	*p* < 0.001
30-day readmission			
Yes	44 (10.2)	7 (7.9)	*p* = 0.499
No	387 (89.8)	82 (92.1)	
Intensive care	12 (2.8)	10 (11.2)	*p* = 0.001
No intensive care	419 (97.2)	79 (88.8)	
Reoperation			
Yes	18 (4.2)	7 (7.9)	*p* = 0.139
No	413 (95.8)	82 (92.1)	

Categorical data are presented as numbers (%) and compared using the chi-square test. Continuous variables are presented as median (Q1–Q3) and compared using the Mann–Whitney *U* test

Compared with elective surgery, emergency patients had a higher ASA classification (*p* = 0.008), more advanced tumour stage (*p* < 0.001), lower BMI (*p* = 0.018) and were more likely to have received chemotherapy for colon cancer (*p* = 0.007). There were no significant differences in age, sex, reoperation rate, or 30-day readmission between groups. Emergency surgery was associated with a longer hospital stay (*p* < 0.001), a higher proportion of open procedures (*p* < 0.001) and increased need for ICU treatment (*p* = 0.001) (Table 1).

### Cost analysis

Hospital costs generally increased with higher Clavien-Dindo grade in both elective and emergency surgery, with lower costs observed for grade V complications. Among patients with no or minor postoperative complications, the median total hospital cost was €9809 (€8614–€11,512) following elective surgery and €12,079 (€10,739–€13,546) following emergency surgery, *p* < 0.001. Among patients with postoperative complications, median total hospital costs were significantly higher after emergency resection at €19,655 (€13,918–€27,134) compared with elective resection at €14,820 (€12,097–€18,862), *p* = 0.004. 

As shown in Tables 2 and 3, total hospital costs generally increased with higher Clavien-Dindo grade across all predefined healthcare categories, in both elective and emergency surgery. However, this pattern was not observed for grade V complications.

**Table 2:** Hospital costs according to postoperative complication severity graded by Clavien-Dindo classification in elective colon cancer surgery

	No or minor complications CD 0–I, *n* = 280	CD II, *n* = 114	CD III, *n* = 21	CD IV, *n* = 11	CD V,* n* = 5	*p* value
Operating room	1302 (1005–1780)	1354 (1107–1776)	1767 (1010–2474)	1651 (1338–3179)	1083 (744–1216)	0.006
Anaesthesia and postoperative unit care	3406 (2971–3892)	3393 (3073–4181)	5598 (3517–6 966)	5356 (4083–9524)	3305 (3144–3581)	<0.001
ICU	0 (0–0)	0 (0–0)	0 (0–0)	7981 (3307–11,047)	0 (0–7879)	<0.001
Laboratory and radiologic expense	925 (801–1005)	1292 (1014–1717)	1349 (946–2753)	2128 (1655–2562)	1066 (848–1806)	<0.001
Medications and material products	421 (329–556)	765 (521–1020)	871 (519–1480)	1544 (885–2459)	732 (2–1214)	<0.001
Staff salaries	2681 (2076–3439)	5010 (3867–6681)	5880 (3290–12,332)	8613 (5080–15,064)	4832 (111–9153)	<0.001
Other costs*	920 (725–1225)	1743 (1273–2337)	2449 (1343–4770)	3507 (1526–4762)	1517 (28–3279)	<0.001
Total costs	9809 (8614–11,512)	14,065 (11,572–16,151)	18,463 (13,366–30,698)	26,307 (22,684–47,893)	14,931 (12,077–19,732)	<0.001

Continuous data are presented as median (Q1–Q3) in 2024 euros (€)

Differences between Clavien-Dindo groups were analysed using the Kruskal-Wallis test

*Premises-related expenses and emergency department utilization

**Table 3:** Hospital costs according to postoperative complication severity graded by Clavien-Dindo classification in emergency colon cancer surgery

	No or minor complications CD 0–I,* n* = 39	CD II,* n* = 31	CD III,* n* = 5	CD IV,* n* = 6	CD V,* n* = 8	*p* value
Operating room	1202 (922–1448)	1640 (1170–2029)	2079 (1492–2940)	2503 (1531–5196)	1460 (1237–1810)	<0.001
Anaesthesia and postoperative unit care	3149 (2769–3676)	3765 (3508–4249)	5461 (4357–7100)	6329 (3714–10,805)	3190 (2676–4400)	<0.001
ICU	0 (0–0)	0 (0–0)	0 (0–0)	18,514 (4456–40,887)	885 (0–4343)	<0.001
Laboratory and radiologic expense	1496 (1245–1700)	1576 (1161–2020)	2737 (1655–171)	2749 (2028–6920)	1281 (909–2104)	0.002
Medications and material products	590 (431–718)	1003 (665–1401)	1857 (902–2141)	1818 (1347–3100)	464 (115–737)	<0.001
Staff salaries	3944 (3398–4952)	6816 (5106–8708)	11,775 (7652–14,249)	11,357 (8928–21,406)	2908 (1449–5362)	<0.001
Other costs*	1530 (1147–1845)	2418 (1481–3654)	4350 (2506–5410)	3849 (3073–6456)	910 (474–1820)	<0.001
Total costs	12,079 (10,739–13,546)	18,290 (14,072–21,516)	31,133 (19,463–33,294)	45,274 (25,812–107,051)	11,617 (7533–24,028)	<0.001

Continuous data are presented as median (Q1–Q3) in 2024 euros (€)

Differences between Clavien-Dindo groups were analysed using the Kruskal-Wallis test

*Premises-related expenses and emergency department utilization

The highest costs were observed among patients with Clavien-Dindo grade IV complications, with median total costs of €26,307 (€22,684–€47,893) following elective surgery and €45,274 (€25,812–€107,051) following emergency surgery. In both groups, total costs associated with Clavien-Dindo grade V complications were lower than those observed for grade III and IV complications, respectively. In the emergency cohort, grade V complications were also associated with lower total costs than grade II complications and the no or minor complication group. The lower observed costs for grade V complications likely reflect a shorter period for hospital resource accumulation due to death, rather than lower resource intensity of care.

The median duration of the index hospital admission was 9 days (IQR 7–13), with 21 patients (4.0%) having an index admission exceeding 30 days. Fifty-eight patients (11.2%) had a second clinically related hospital admission, and three patients (0.6%) had a third admission. The median duration of second admissions was 5.5 days (IQR 3–10).

### Adjusted costs and excess costs according to complication severity and type

After adjustment for tumour stage, ASA classification, surgical technique, sex and age in the elective model, and for tumour stage, ASA classification, sex and age in the emergency model, increasing complication severity remained significantly associated with higher costs in the generalized linear model analysis. Adjusted mean costs generally increased with higher Clavien-Dindo grade in both elective and emergency surgery, although grade V did not follow this pattern. In elective surgery, Clavien-Dindo grade IV complications were associated with more than a threefold increase in costs (cost ratio 3.21), whereas emergency patients demonstrated an almost fivefold increase in costs (cost ratio 4.86) compared with patients with no or minor complications. This estimate was based on six patients with CD IV complications and should be interpreted cautiously because of the small subgroup and the number of parameters in the emergency model. Results of the parsimonious models are presented in Table 4.

**Table 4:** Multivariable generalized linear model (GLM) analysis of total hospital costs in elective and emergency surgery

	Elective resections, *n* = 431	*p* value		Emergency resections, *n* = 89	*p* value
	Cost ratio, 95% CI			Cost ratio, 95% CI	
Complication severity (Clavien-Dindo classification)					
No or minor complications					
(CD 0–I)	Ref	NA		Ref	NA
CDII	1.40 (1.31–1.51)	<0.001		1.56 (1.29–1.88)	<0.001
CDIII	2.11 (1.70–2.60)	<0.001		2.05 (1.53–2.76)	<0.001
CDIV	3.21 (2.49–4.13)	<0.001		4.86 (2.84–8.31)	<0.001
CDV	1.50 (1.13–2.00)	0.005		1.12 (0.74–1.68)	0.596
Tumour stage					
Stage I	Ref	NA		Ref	NA
Stage II	1.10 (1.03–1.18)	0.005		1.34 (1.11–1.62)	0.002
Stage III	1.04 (0.97–1.12)	0.241		1.21 (1.04–1.39)	0.011
Stage IV	1.09 (0.89–1.32)	0.401		1.10 (0.92–1.32)	0.307
ASA classification					
ASA I	Ref	NA		Ref	NA
ASA II	0.97 (0.89–1.06)	0.487		0.92 (0.72–1.18)	0.519
ASA III	0.99 (0.90–1.10)	0.882		1.04 (0.79–1.37)	0.769
ASA IV	0.87 (0.60–1.26)	0.465		0.91 (0.53–1.56)	0.727
Surgical technique^†^					
Laparoscopic Open	Ref 1.16 (1.09–1.23)	NA <0.001		-	-
**Sex**					
Female	Ref		Ref		
Male	1.05 (0.99–1.11)	0.099	1.04 (0.88–1.22)		0.666
**Age**					
Age per year	1.004 (1.001–1.007)	0.019		0.998 (0.992–1.004)	0.528

Results are presented as cost ratios (exp(B)) with 95% confidence intervals and *p* values. Models were fitted using a gamma distribution with a log link. The scale parameter was estimated using maximum likelihood, and robust covariance estimates were used

^†^Surgical technique was retained in the elective model but excluded from the emergency model because only 3 of 89 emergency patients underwent laparoscopic resection

**Fig. 1:**
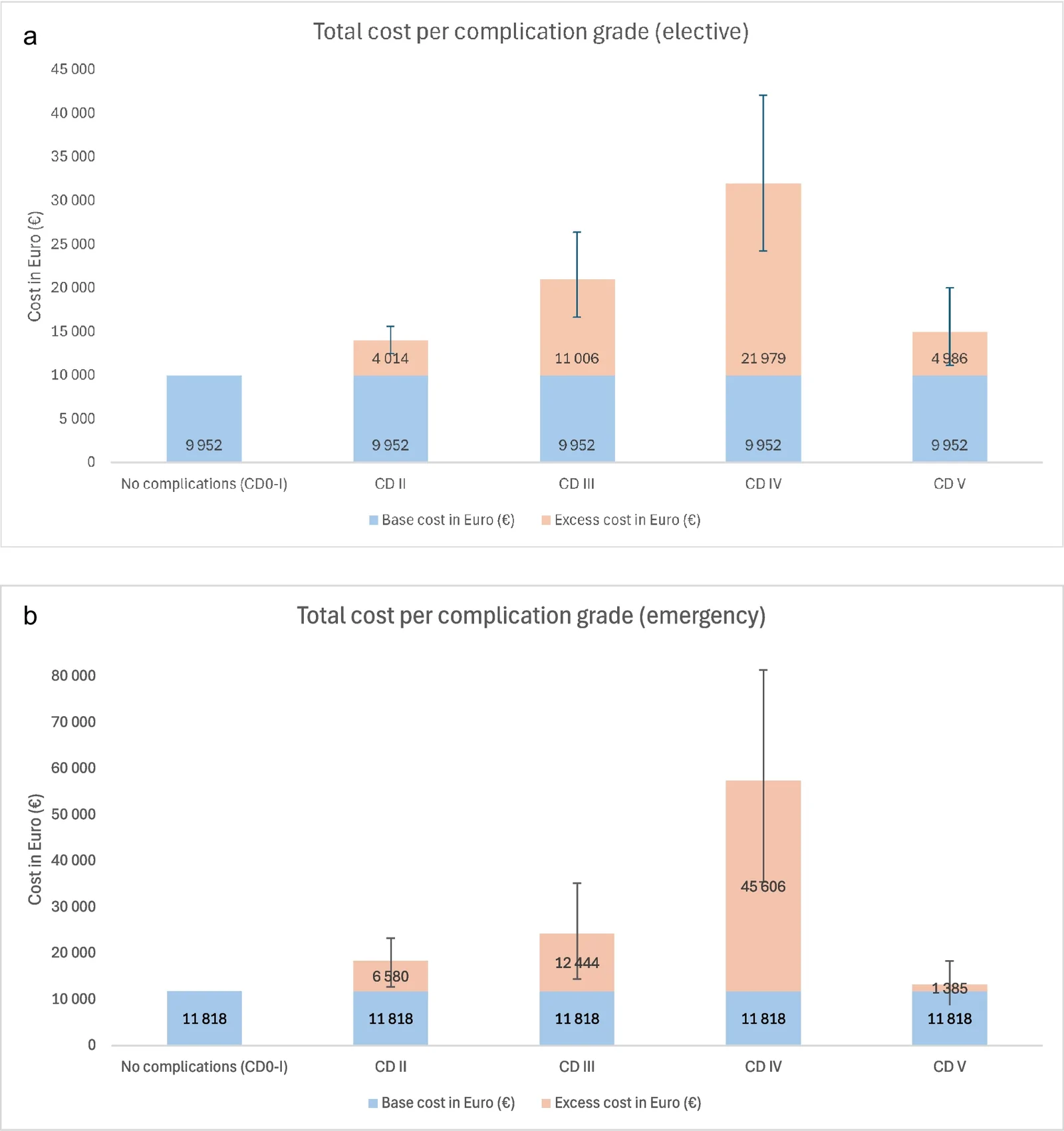
Total hospital costs stratified according to postoperative complication severity in elective (**a**) and emergency (**b**) colon cancer surgery. The blue bars represent the estimated baseline cost for patients with no or minor postoperative complications, while the orange bars represent the adjusted excess costs associated with increasing Clavien-Dindo (CD) grades. Error bars indicate 95% confidence intervals for the excess cost estimates

Adjusted excess costs generally increased from Clavien-Dindo grade 0–I through Clavien-Dindo grade IV, in both elective and emergency surgery, whereas grade V did not follow this pattern (Fig. 1a and b). The largest excess costs were observed among patients with Clavien-Dindo grade IV complications, with a more pronounced increase in emergency surgery. This association was less consistent for grade V complications, for which estimates were based on few patients and should therefore be interpreted with caution, particularly in the emergency cohort.

**Fig. 2:**
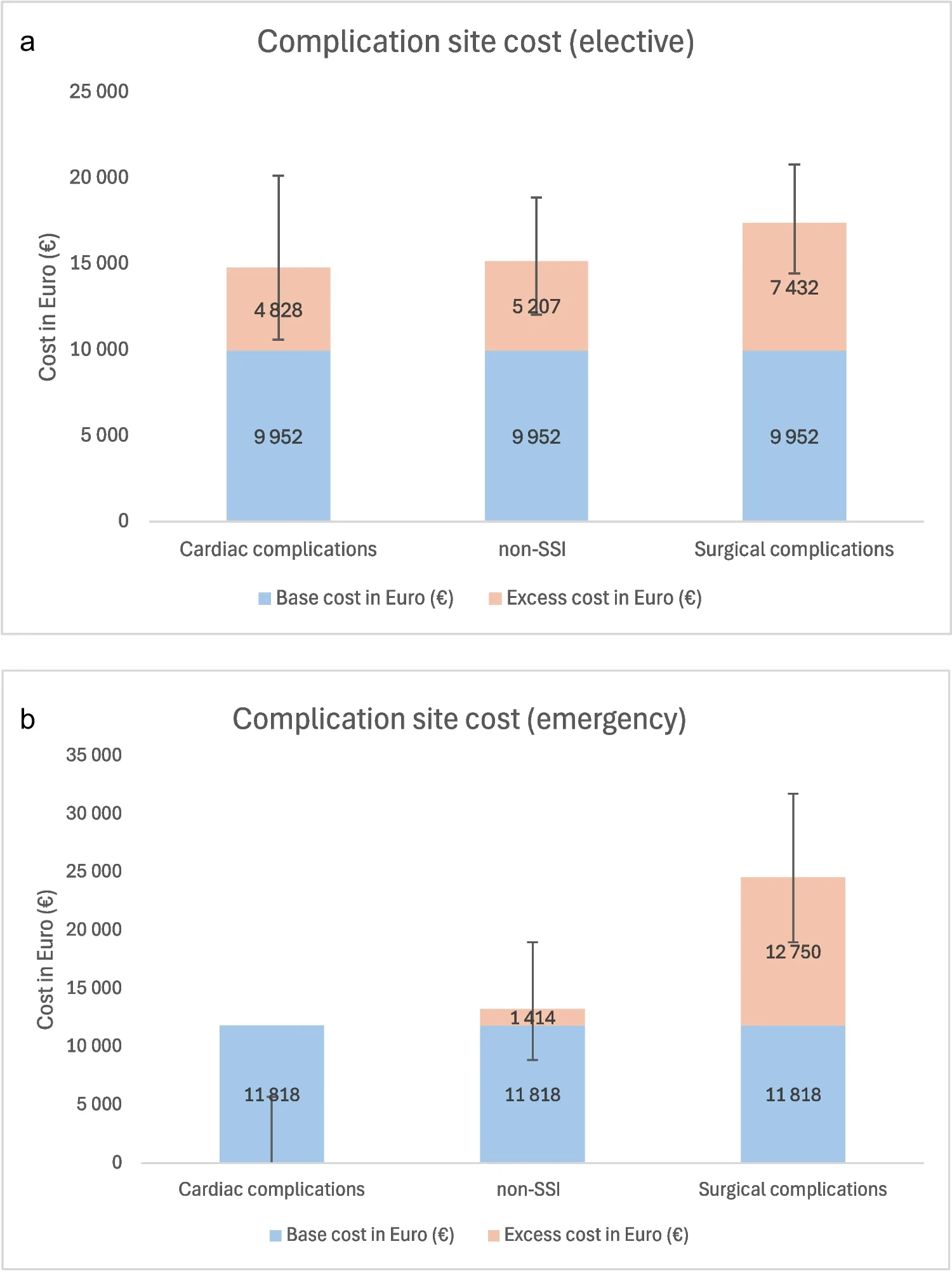
Total hospital costs according to postoperative complication category in elective (**a**) and emergency (**b**) colon cancer surgery. Complication categories were cardiac, non-surgical-site infections (non-SSI), and surgical complications. Bars represent total mean costs divided into baseline costs for patients with no or minor complications (blue) and excess costs associated with postoperative complications within each complication category (orange). Baseline costs are model-estimated costs for patients with no or minor complications within the respective surgical group. Error bars indicate 95% confidence intervals for excess costs. No error bar is shown for cardiac complications in the emergency group because the estimated excess cost was negative (−€24, 95% CI (€−5968–€9002))

Figure 2a and b illustrates adjusted excess costs according to complication type. In elective surgery, all complication categories were associated with increased costs, with the greatest excess costs observed for surgical complications. In emergency surgery, surgical complications remained strongly associated with increased costs, whereas cardiac and non-SSI complications were not independently associated with significantly increased costs.

## Discussion

This retrospective single-centre study used patient-level hospital cost data and adjusted generalized linear models to evaluate complication-associated costs across Clavien-Dindo grades following elective and emergency colon cancer surgery. We demonstrated an overall association between complication severity and higher hospital costs. Emergency surgery was associated with higher and more variable costs, reflecting greater clinical complexity. Surgical complications were the main drivers of excess costs in both elective and emergency surgery, whereas cardiac and non-SSI complications showed less consistent associations with increased costs.

Postoperative complications are major drivers of hospital costs following colon cancer surgery. However, previous studies have applied heterogeneous methodological approaches, often focusing on selected complication types or surgical techniques rather than overall complication burden or severity. Systematic reviews [7, 15] showed that anastomotic leakage and surgical site infections are among the complications associated with the highest financial burden, highlighting the economic impact of clinically relevant adverse events. Large population-based studies have similarly demonstrated substantially higher hospital costs, prolonged hospital stay, and increased mortality among patients developing anastomotic leakage [16, 17]. However, analyses of isolated complications have limited clinical relevance, as patients frequently experience multiple concurrent complications and resource use is better captured by overall morbidity burden than by single events.

In this study, complications were classified using the Clavien-Dindo classification, a standardized and widely accepted framework that enables assessment of complication severity across the different grades [8]. Although only a limited number of cost analyses in colorectal cancer surgery have applied this classification, previous evidence has demonstrated higher costs with increasing Clavien-Dindo grade [2, 4], consistent with our findings. This supports an overall association between complication severity and resource use, although this pattern was not monotonic for grade V complications. The lower costs associated with Clavien-Dindo grade V complications likely reflect a shorter time window for resource accumulation rather than a lower resource intensity of care.

In analyses stratified by complication type, surgical complications were associated with the highest excess costs in both elective and emergency surgery. In emergency surgery, cardiac and non-SSI complications were not independently associated with significantly increased costs in the adjusted model. However, overlap between complication categories should be acknowledged, as patients may experience multiple concurrent events, which limits attribution to individual complication types.

Surgical complications are well-established drivers of cost following colon cancer surgery. Superficial surgical site infections are associated with lower excess costs compared with deep infections, whereas anastomotic leakage is associated with particularly high costs [15]. A graded increase in costs has been observed with increasing severity of surgical site infection [3], while the Clavien-Dindo classification similarly reflects increasing clinical severity and resource needs across grades. Consistently, a small subgroup of patients with severe complications accounts for a disproportionate share of total hospital costs. In the present study, the adjusted excess costs associated with complications were substantial relative to the model-estimated mean total cost for patients with no or minor complications. For CD II complications, excess costs corresponded to 40% of the baseline cost in elective surgery (€4014 vs €9952) and 56% in emergency surgery (€6580 vs €11,818). For CD III complications, the corresponding excess costs slightly exceeded the baseline cost, amounting to 111% in elective surgery (€11,006 vs €9,952) and 105% in emergency surgery (€12,444 vs €11,818). Previous studies have similarly demonstrated an association between increasing Clavien-Dindo grade and higher healthcare costs after colorectal surgery. Widmar et al. [18] showed that lower-grade complications contribute substantially to post-discharge healthcare utilization, while Rintala et al. [19] recently confirmed a progressive increase in 90-day hospital costs across Clavien-Dindo grades and particularly high costs associated with anastomotic leakage. High-grade complications, particularly Clavien-Dindo IV events, are likely driven by intensive care admission, organ support, reinterventions and prolonged hospital stay, often in the context of multiple concurrent complications [20].

When stratified by surgical setting, important differences were observed between elective and emergency surgery [21]. In the present cohort, 17.1% of resections were classified as emergency procedures, compared with approximately 11% nationally. This difference may partly reflect differences in how emergency surgery is defined, as well as local referral patterns and case mix. Previous cost analyses have predominantly focused on elective patients [5, 6, 22, 23], whereas some studies have included both elective and emergency resections in their analyses [3, 4]. Given the substantial differences in baseline physiology, disease stage, and perioperative risk, elective and emergency patients are not comparable with respect to resource utilization. The higher proportion of patients receiving chemotherapy for colon cancer in the emergency group may partly reflect the more advanced tumour stage observed in this group, although the reasons for subsequent emergency presentation cannot be determined from the present data. Furthermore, the underlying indication for emergency surgery may itself contribute to increased resource utilization independently of postoperative complications, and these factors cannot be fully disentangled in an observational study. Nevertheless, emergency surgery was associated with both higher and more variable hospital costs. These differences likely reflect the combined effects of emergency presentation, greater baseline clinical complexity, and postoperative morbidity [24, 25]. In contrast, elective surgery allows for preoperative optimization and structured perioperative pathways, which may reduce both complication rates and variability in resource utilization. Although emergency patients experienced more severe postoperative complications, 30-day readmission rates were numerically higher among elective patients. This may partly reflect differences in the timing of complication detection, as severe complications in emergency patients may be identified during the index admission before discharge.

Together, these findings reinforce postoperative complications as a major and potentially modifiable driver of hospital costs. Interventions aimed at reducing complication rates and accelerating recovery, including enhanced recovery pathways, minimally invasive surgical techniques, and early recognition of postoperative deterioration, may therefore provide both clinical and economic benefits. Furthermore, given that a substantial proportion of colon cancer resections are still performed urgently, strategies aimed at reducing emergency presentations or enabling more patients to undergo planned elective resection may further reduce both morbidity and healthcare expenditures [26]. By identifying the complication profiles and surgical settings associated with the greatest economic burden, our findings may help inform quality improvement initiatives and support prioritization of preventive strategies and healthcare resource allocation.

A major strength of this study was the use of a patient-level micro-costing approach, with direct hospital costs estimated in close collaboration with a health economist. Resource utilization was assessed at the individual patient level, with multiple cost components assigned directly to each patient using the institutional cost-accounting system. In addition, generalized linear models enabled adjusted cost estimates while accounting for potential confounding factors and the skewed distribution of healthcare cost data. Importantly, complications were systematically identified through detailed review of individual medical records in combination with registry data, allowing a more complete ascertainment of postoperative events than registry-based data alone. This comprehensive ascertainment may partly explain the higher complication rates observed in our cohort compared with national estimates based primarily on registry data.

Another strength was the separate analysis of elective and emergency surgery. To our knowledge, few previous studies have directly compared complication-associated costs between these surgical settings, providing new insight into the economic burden associated with emergency colon cancer surgery. Finally, postoperative complications were classified according to the Clavien-Dindo classification, allowing evaluation of the association between complication severity and hospital costs. This enabled a clinically meaningful evaluation of postoperative morbidity and its economic impact.

Several limitations should be acknowledged. First, the single-centre retrospective design may limit generalizability. The relatively high proportion of open surgery in the elective cohort (71.5%) should also be considered when interpreting the cost estimates and may limit their generalizability to contemporary settings with greater use of minimally invasive surgery. Second, although the micro-costing approach increases precision, cost structures and accounting systems may differ between healthcare systems, potentially limiting external validity. Third, retrospective assessment of complications may increase the risk of underreporting minor postoperative events, particularly lower-grade complications. Fourth, higher Clavien-Dindo grades were relatively rare, particularly in the emergency cohort, resulting in limited numbers within individual grades and reduced statistical precision of the corresponding estimates. Fifth, only direct costs were included, and the total societal economic burden associated with postoperative complications is therefore likely underestimated. Because costs were recorded for complete hospital admissions rather than individual hospital days, costs could not be truncated at 30 or 90 days after surgery. The exact time from index surgery to the final cost-bearing hospital encounter could therefore not be determined from the available cost data. The absence of a predefined maximum cost-accrual window should therefore be considered when interpreting the results and when comparing them with studies using a predefined 90-day cost horizon. The emergency cohort was heterogeneous, comprising patients with different indications for surgery. The definition of emergency surgery as resection within 48 h of acute hospital admission may have resulted in some misclassification compared with definitions based on clinical indication rather than timing, particularly in patients with an acute presentation who underwent initial conservative management before surgery. The limited sample size precluded meaningful subgroup analyses, and future studies should investigate whether specific emergency presentations are associated with different postoperative costs and outcomes. Finally, long-term oncological outcomes and subsequent cancer treatment costs were not assessed in the present study. Future analyses integrating cost data with oncological outcomes are warranted to evaluate the broader consequences of postoperative complications.

## Conclusion

Postoperative complications after colon cancer surgery were associated with substantially higher hospital costs, with the greatest excess cost observed for severe complications, particularly Clavien-Dindo grade IV. Grade V did not follow this pattern, likely reflecting a shorter period of cost accumulation due to death. Observed hospital costs were generally higher and more variable following emergency than elective surgery.

A small group of patients with severe complications appears to drive a large proportion of total hospital costs, underscoring complications as a modifiable cost driver. Efforts to reduce emergency presentations and optimize perioperative pathways may therefore have both clinical and economic benefits.

## Supplementary Information

Below is the link to the electronic supplementary material.

Supplementary file1 (DOCX 33 kb)

## Data Availability

The data underlying this study were obtained from regional healthcare databases and are not publicly available due to patient confidentiality and Swedish data protection legislation. Access to de-identified data may be granted upon reasonable request, subject to approval by the relevant ethical review authority and the data-holding institution.

## Declarations

### **Previous presentation**

This paper has not been presented previously.
